# A Novel Method for Designing General Window Functions with Flexible Spectral Characteristics

**DOI:** 10.3390/s18093081

**Published:** 2018-09-13

**Authors:** Yinghao Sun, Quanhua Liu, Jinjian Cai, Teng Long

**Affiliations:** Key Laboratory of Electronic and Information Technology in Satellite Navigation, Beijing Institute of Technology, Beijing 100081, China; bitersunyinghao@gmail.com (Y.S.); caijinjian@bit.edu.cn (J.C.); longteng@bit.edu.cn (T.L.)

**Keywords:** window functions, spectral characteristics, mainlobe width (MW), peak sidelobe level (PSL), sidelobe fall-off rate (SLFOR), steerable sidelobes

## Abstract

In the field of sensor signal processing, windows are time-/frequency-domain weighting functions that are widely applied to reduce the well-known Gibbs oscillations. Conventional methods generally control the spectral characteristics of windows by adjusting several of the parameters of closed-form expressions. Designers must make trade-offs among the mainlobe width (MW), the peak sidelobe level (PSL), and sometimes the sidelobe fall-off rate (SLFOR) of windows by carefully adjusting these parameters. Generally, not all sidelobes need to be suppressed in specified applications. In this paper, a novel method, i.e., the inverse of the shaped output using the cyclic algorithm (ISO-CA), for designing window functions with flexible spectral characteristics is proposed. Simulations are conducted to test the effectiveness, flexibility and versatility of the method. Some experiments based on real measured data are also presented to demonstrate the practicability. The results show that the window functions generated using the cyclic algorithm (CA) yield better performance overall than the windows of conventional methods, achieving a narrower MW, a lower PSL, and a controllable SLFOR. In addition, steerable sidelobes over specified regions can be acquired both easily and flexibly while maintaining the original properties of the initial window as much as possible.

## 1. Introduction

Window functions are widely used to reduce the well-known Gibbs oscillations resulting from the truncation of a Fourier series [1]. This century-old technology is employed in many applications in the sensor signal processing field [2,3,4]. In most of these applications, a window function is expected to behave as a Dirac function in the frequency domain, that is, to concentrate all the spectral power into an extremely narrow band with zero sidelobes. However, this behaviour exists neither in theory nor in practice. A window function always has a mainlobe width and sidelobes. As stated in [5], no window is suitable for all cases, and one should be selected according to the requirements of a particular application. Thus, a method for designing window functions with flexible spectral characteristics is greatly needed. Commonly used spectral characteristics of a window function include the mainlobe width (MW), the peak sidelobe level (PSL), and the sidelobe fall-off rate (SLFOR), which are closely related to the resolution and spectrum leakage. In addition, steerable sidelobes over specified regions are needed in some applications.

Due to the importance and significance of window functions, the literature on this topic is unsurprisingly extensive (e.g., see [1,2,3,4,5,6,7,8,9,10,11,12,13,14,15,16,17,18,19,20,21,22,23,24,25] and the references therein). Traditional windows, including triangular, Hamming, Hanning, Blackman, and other well-known windows, aim at smoothing the truncated impulse response in the time domain, resulting in lower sidelobes at the expense of a wider mainlobe [1]. However, the spectral characteristics of these windows cannot be controlled because of the fixed expressions. Scholars later presented many window families with adjustable parameters, and trade-offs can be made to control different properties [6,7,8,9,10,11,12,13,14,15,16,17]. In recent years, a triangular self-convolution window has been obtained by convolving the triangular window as presented in [18], and it exhibits a good deal of lower PSL and higher SLFOR. Its applications in harmonic estimation are discussed in [19,20]. In [21], optimized trapezoid convolution windows are proposed, leading to a narrow main lobe width but retaining a low PSL as well as a fast SLFOR. In particular, Tseng provided a novel window function with steerable sidelobes with which a deep dip can be steered to any frequency, effectively improving the detectability of a small tone without degrading its resolvability [22,23]. Several windows combining steerable sidelobes and other properties were presented in [24,25]. However, the parameters of the expressions proposed above should be carefully chosen and repeatedly tuned to obtain satisfactory performance, which is complicated and unintuitive. Moreover, to the best of our knowledge, none of these methods can control all spectral characteristics of window functions at the same time. Window functions combining steerable sidelobes with controllable spectral characteristics have not been proposed to date. In addition, in a particular case, window functions should be specially designed according to specific applications; however, the methods mentioned above are not very applicable. For example, in the radar signal processing field, the complex linear frequency modulation (LFM) signal has a rectangle-like shape in the frequency domain. The common pulse compression method for echoes of the LFM signal is usually carried out by using the matched filter (MF) method. The final step of the MF method is to conduct an inverse discrete Fourier transform (IDFT) on a rectangle-like spectrum. The output of the MF in the time domain has a high PSL. Thus, window functions in the frequency domain are needed in the final step. Obviously, the output of the MF is related to not only the window functions but also the spectrum of the LFM. Hence, many properties of the window functions cannot be acquired by using the existing methods, which are based on rectangular windows by default, and partial or total effectiveness will be lost in these applications.

In other recent research, Stoica P. proposed many algorithms, including cyclic algorithms (CAs), CA-pruned (CAP), CA-new (CAN), and weighted CAN (We CAN), for applications in unimodular phase-coded waveform design [26]. The designed waveforms have ultra-low range sidelobes over specified regions after pulse compression. Then, an FFT-based CA for designing a unimodular sequence train with low central and recurrent autocorrelation sidelobes is proposed in [27]. Some numerical properties of the CA for the estimation of the parameters of a multinomial model is presented in [28]. Performance of the CA is also compared with some of the best available optimization algorithms. It should be noted that CA is not a technical term but simply a name to describe the iteration procedure, and all CAs in [26,27,28] and this paper are different in the details. In addition, the convergence analysis and the recursion formula in these papers are not given. Interestingly, CA is a good iteration idea for solving optimization problems and may be well applied in the window-design field.

In this paper, we aim to propose a method for designing general window functions in which all spectral characteristics and steerable sidelobes can be easily and flexibly controlled. In addition, these windows should be adaptable to different cases such as non-rectangular applications. Thus, a novel method, i.e., the inverse of the shaped output using the cyclic algorithm (ISO-CA), for designing general window functions with flexible spectral characteristics is proposed. The ISO-CA is based on only the fast Fourier transform (FFT) and the inverse FFT (IFFT) and thus can accelerate the iterative procedure. The recursion formula is also provided, and the convergence of the iterations is proven. The results of experiments and simulations show that flexible spectral characteristics, including the MW, PSL, SLFOR, and steerable sidelobes, for the designed window function can be easily obtained using the proposed CA method. In addition, it is more flexible and intuitive for designers to use instead of choosing and adjusting several parameters. Moreover, any existing window with good properties can be chosen as an initial window function of the algorithm so that the generated CA window function will have both the properties of the initial window and the designated spectral characteristics. Thus, using the proposed method, windows can be specially designed for specific applications to which existing methods would not apply.

This paper is organized as follows. In Section 2, the model derivation is introduced. Some simulations and experiments are presented in Section 3. Section 4 concludes this paper. The convergence analysis of the ISO-CA is provided in the Appendix.

## 2. Model Derivation

Window functions are selected principally based on their spectral characteristics. The ISO-CA proposed in this paper is a novel method for designing general window functions with flexible spectral characteristics, for which an existing window should be chosen as the initial input. Here, we choose the rectangular window function as an example:

(1) $$w={\left[1,1,\cdots ,1\right]}^{T},$$

where ${\left[\cdot \right]}^{T}$ denotes the transpose operator. The length of the rectangular window is set to *N*. The rectangular window in the frequency domain can be expressed as:

(2) $$W=F_{M}{\left[\begin{array}{c} w\\ 0 \end{array}\right]}_{M\times 1},$$

where $F_{M}$ is the *M*-point DFT matrix:

(3) $$F_{M}=\left[\begin{array}{cccc} 1 & 1 & \cdots & 1\\ 1 & \Omega_{M}^{1} & \cdots & \Omega_{M}^{M-1}\\ \vdots & \vdots & \ddots & \vdots \\ 1 & \Omega_{M}^{M-1} & \cdots & \Omega_{M}^{\left(M-1\right)\left(M-1\right)} \end{array}\right],\Omega_{M}^{k}=\exp\left(-j\frac{2\pi }{M}k\right).$$

We pad $\left(M-N\right)$ zeros at the end of the window in (2) to decrease the well-known picket fence effects of the DFT caused by the truncation and discreteness. In addition, *N* and *M* are usually set to even numbers to maintain the “even” property of the window [5]. For convenience, we can rewrite (2) as:

(4) $$W=F_{MN}w,$$

where $F_{MN}$ denotes the first *N* columns of $F_{M}$.

Then, we define the spectral characteristics of the window functions. For the PSL:

(5) $$\mathrm{PSL}\left(\mathrm{dB}\right)=10\cdot \mathrm{lo}g_{10}\left(\frac{\max\left\{{\left|W\left(k\right)\right|}^{2}\right\}}{{\left|W\left(0\right)\right|}^{2}}\right),k\in \mathrm{sidelobe}\;\mathrm{area},$$

which denotes the maximal level of the sidelobes or the sidelobe attenuation. Note that steerable sidelobes can be obtained by the proposed method. The ISL over a specified interval $\left[a,b\right]$ is defined as follows:

(6) $$\underset{a\to b}{\mathrm{ISL}}\left(\mathrm{dB}\right)=10\cdot \log_{10}\left(\frac{\sum_{k=a}^{b}{\left|W\left(k\right)\right|}^{2}}{{\left|W\left(0\right)\right|}^{2}}\right),a,b\in \mathrm{sidelobe}\;\mathrm{area}.$$

The SLFOR (dB/oct) denotes how many dB the sidelobes decrease by per octave, which measures the sidelobe fall-off rate. For the MW, the width of the mainlobe at 3 dB below the mainlobe peak is commonly used [1]. Windows with different spectral characteristics are needed in different situations. Thus, a method for designing window functions that can control all these spectral characteristics is greatly needed.

Now, we design a mask vector:

(7) $$\begin{array}{c} \Gamma ={\left[\Gamma \left(0\right),\Gamma \left(1\right),\cdots ,\Gamma \left(M-1\right)\right]}^{T}\\ \Gamma \left(p\right)=1,p\in \mathrm{mainlobe}\;\mathrm{area}\\ \Gamma \left(q\right)\in \left[0,1\right],q\in \mathrm{sidelobe}\;\mathrm{area} \end{array}$$

to shape the output w and obtain the desired spectral characteristics:

(8) $$W_{\Gamma }=\Gamma ⊙W,$$

where ⊙ is the Hadamard (element-wise) product. Note that window functions are usually real, even and nonnegative in the time domain [5]. Thus, Γ should satisfy:

(9) $$\Gamma \left(i\right)=\Gamma \left(M-1-i\right),\forall i\in \left[0,M-1\right]$$

to maintain these properties according to the DFT theorem.

*q* and $\Gamma \left(q\right)$ can be designed and adjusted easily and flexibly according to different requirements of the PSL, SLFOR, and steerable sidelobes. The advantage of the mask vector Γ is that we can change only the spectral characteristics of interest while maintaining the others as much as possible.

The problem now is how to design a window function $w_{\Gamma }$ such that:

(10) $$F_{MN}w_{\Gamma }=W_{\Gamma }.$$

Equation (10) is actually an overdetermined matrix equation with *M* equations and *N* unknowns, where M≫N generally. Only in very special circumstances does an overdetermined equation have an accurate solution, but a least squares solution can always be acquired by calculating the left inverse of the matrix $F_{MN}$:

(11) $${\hat{w}}_{\Gamma }=F_{MN}^{†}W_{\Gamma },$$

where $F_{MN}^{†}={\left(F_{MN}^{H}F_{MN}\right)}^{-1}F_{MN}^{H}$, and ${\left[\cdot \right]}^{H}$ denotes the conjugate transpose or the Hermitian transpose operator. We know that matrix $F_{MN}$ is part of $F_{M}$, the columns of which are orthonormal base vectors:

(12) $$F_{MN}^{H}F_{MN}=M\cdot I_{NN},$$

where $I_{NN}$ is an N×N unit matrix. Thus, we can obtain:

(13) $$F_{MN}^{†}=\frac{1}{M}F_{MN}^{H}.$$

According to the DFT theorem, the IDFT matrix satisfies:

(14) $$F_{M}^{-1}=\frac{1}{M}F_{M}^{H}=\left[\begin{array}{c} \frac{1}{M}F_{MN}^{H}\\ \vdots \end{array}\right]=\left[\begin{array}{c} F_{MN}^{†}\\ \vdots \end{array}\right]$$

Equation (14) is an interesting conclusion. It indicates that we can replace the left inverse calculation with the IFFT and simply take the first *N* values of the result to obtain ${\hat{w}}_{\Gamma }$.

We now obtain a simple closed-form solution to the expected window function. However, we may not acquire the anticipatory spectral characteristic as expressed in (8) because of the natural errors of the least squares solution. To compensate for these errors of solution (11), the ISO-CA is proposed.

The solution provided in (11) has only some of the features of the desired spectral characteristic as expressed in (8). Thus, the idea is to enhance the features via further iterations. The calculated ${\hat{w}}_{\Gamma }$ is chosen as the new initial window function w, and then the procedure above is repeated to allow the spectral characteristics to gradually fit the mask vector Γ.

The ISO-CA can be summarized as Algorithm 1 shows.
**Algorithm 1** ISO-CA Set initial w and Γ; **repeat**  1. Calculate the *M*-point FFT of w to obtain W (Equation (4));  2. Update the mask vector Γ and calculate: $W_{\Gamma }=\Gamma ⊙W$ (Equation (8));  3. Calculate the *M*-point IFFT of $W_{\Gamma }$, and take the first *N* values to obtain ${\hat{w}}_{\Gamma }$ (Equation (14));  4. Replace w with ${\hat{w}}_{\Gamma }$; **until** the stop criterion is met.

It can be seen from the flow that each iteration contains: calculating the FFT of the window in the time domain, shaping the output to obtain the desired spectral characteristics, inverting the shaped output using the IFFT, and replacing the old window with the new one. Thus, we call it the inverse of the shaped output using the ISO-CA method. The iteration of the ISO-CA has linear convergence rates. The recursion formula and derivation are presented in the Appendix. This algorithm procedure shows that only the FFT, the Hadamard product, and the IFFT are used in each iteration without complex computations. Thus, by taking advantage of the simplicity of the FFT and IFFT, the efficiency of the iteration is greatly improved.

It is possible to create a wide dip centred at a specified frequency without broadening the mainlobe [22]. However, the suppression of the near-sidelobes is obtained at the expense of a broadened mainlobe. Thus, in this case, the data range of *p* in (7) should be updated during the iteration. In addition, to control the spectral characteristics of the window function, the criterion for stopping the iteration can be designed as follows:

(15) $$\mathrm{stop}\;\mathrm{criterion}\left\{\begin{array}{c} i\geq I_{\max}\\ {∥w_{i}-w_{i-1}∥}_{2}\leq \varepsilon_{0}\left(i\geq 2\right)\\ \mathrm{PSL}\left(i\right)\leq \varepsilon_{1}\\ \underset{a\to b}{\mathrm{ISL}}\left(i\right)\leq \varepsilon_{2}\\ \mathrm{SLFOR}\left(i\right)\geq \varepsilon_{3}\\ \mathrm{MW}\left(i\right)\geq \varepsilon_{4} \end{array}\right.$$

where $I_{\max}$ denotes the maximum number of iterations; $\varepsilon_{k},k\in \left[0,4\right]$ is the prescribed tolerance; and $\mathrm{PSL}\left(i\right)$, $\underset{a\to b}{\mathrm{ISL}}\left(i\right)$, $\mathrm{SLFOR}\left(i\right)$, and $\mathrm{MW}\left(i\right)$ denote the respective spectral characteristics of $w_{i}$ in the *i*-th iteration. The first two criteria in (15) are regular iteration stop conditions, where $I_{\max}$ constrains the maximum number of iterations and $\varepsilon_{0}$ denotes the convergence condition. $\varepsilon_{0}$ is set to a very small number that denotes the condition of convergence, and trade-offs can be achieved by setting $\varepsilon_{1}$ to $\varepsilon_{4}$. The remaining four criteria can be designed in any combination to control the spectral characteristics of the generated window function according to the requirement. Typically, $\varepsilon_{0}$ is set as low as possible, and designers must then balance the PSL, SLFOR, MW, and steerable sidelobes by setting $\varepsilon_{1}$ to $\varepsilon_{4}$.

## 3. Simulations and Experiments

In this section, we present some simulations and experiments, which are divided into four main parts, to show the effectiveness, flexibility, versatility and practicability of the proposed method.

### 3.1. Controllable Spectral Characteristics

In this subsection, we generate several window functions using the proposed ISO-CA based on the rectangular window function to compare the spectral characteristics with those of some frequently used windows.

**Figure 1:**
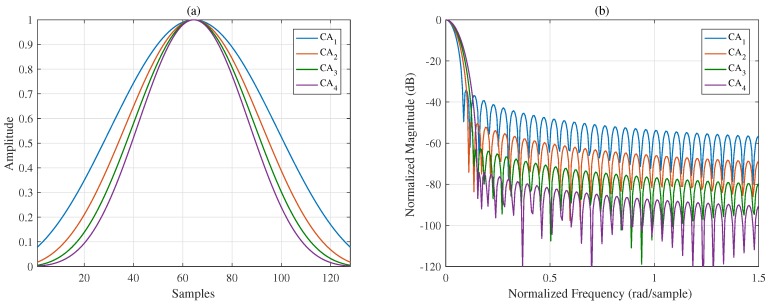
Windows with different MWs and PSLs (**a**) in the time domain and (**b**) in the frequency domain.

**Figure 2:**
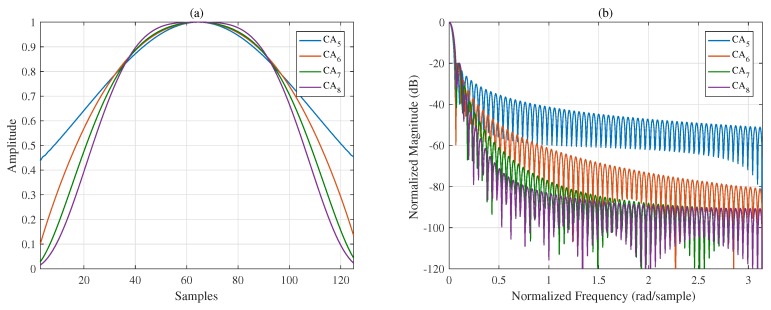
Windows with different SLFORs (**a**) in the time domain and (**b**) in the frequency domain.

To suppress all the sidelobes, let $\Gamma \left(q\right)=0$. The interval $q\in \left[a,b\right]$ of the mask vector Γ in (7) should contain the entire sidelobe region and should be adjusted adaptively in each iteration because of the continually widening MW. The abscissas of the figures in the time domain and frequency domain are discrete samples and the normalized frequency, respectively. Eight CA window functions with different designed spectral characteristics are presented in Figure 1 and Figure 2; the length of each window function is 128 discrete samples. Figure 1a,b shows that trade-offs between the MW and PSL can be made. To better compare the properties of different window functions, the spectral characteristics of the generated CA window functions and other commonly used windows are listed in Table 1. Comparing $CA_{1}$ to $CA_{4}$ with the traditional window functions, when the MWs are the same, the proposed windows provide smaller PSLs. It is not difficult to infer that if the PSLs are the same, the MWs of the proposed windows must be narrower. Additionally, note that the Chebyshev window has the smallest PSL for a given MW with the SLFOR equal to zero [1]. Thus, the proposed windows have lower sidelobes over distant regions, although the PSL is slightly higher than that of the Chebyshev window.

With the fall-off rate of the mask vector Γ controlled, four window functions in the frequency domain with a fixed PSL (−20 dB) and different SLFORs are presented in Figure 2a,b. Figure 1 and Figure 2 show that the spectral characteristics of windows can be controlled flexibly and effectively using the proposed method. In summary, in addition to the flexibility of the proposed method, the window functions generated by the proposed methods yield better results overall, achieving a narrow MW, a small PSL, and a controllable SLFOR.

### 3.2. Steerable Sidelobes

We know that not all the sidelobes need to be suppressed in some applications if prior knowledge is acquired in advance. Thus, steerable sidelobes are needed. As mentioned in the Introduction, several methods for designing window functions with steerable sidelobes have been proposed. Steerable sidelobes were first proposed by Tseng et al. [22,23], for which the IDFT is needed to evaluate the temporal weights. Zhong et al. [24] evaluated explicit expressions of windows with steerable sidelobes using the sum of the cosine terms. However, the improvement in the SLFOR was not sufficiently investigated mathematically. In [25], Magdy combined the objectives of a high SLFOR and many steerable sidelobe dips but seemingly did not consider the PSL and MW.

**Figure 3:**
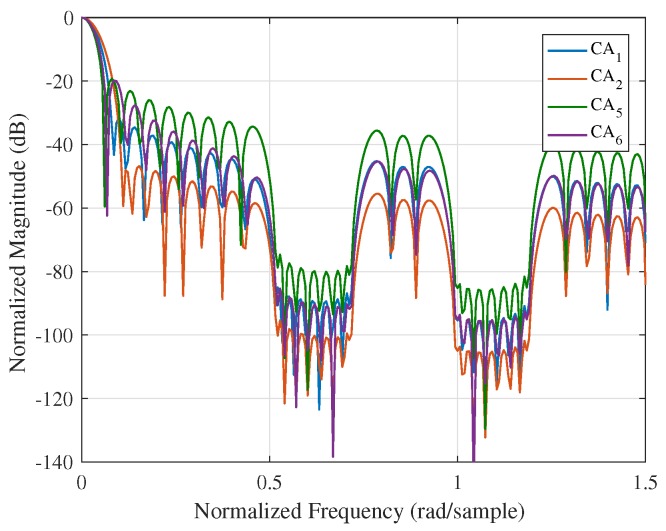
Window functions with steerable sidelobes.

In this paper, steerable sidelobes are easily obtained by making dips in the mask vector. Other spectral characteristics can also be effectively controlled by designing the stop criterion of the iteration. To demonstrate the performance of the proposed method, four typical CA windows, i.e., $CA_{1}$, $CA_{2}$, $CA_{5}$, and $CA_{6}$, are chosen as the initial windows of the algorithm. Windows with steerable sidelobes are presented in Figure 3. The intervals $q\in \left[0.5,0.7\right]\cup \left[1.0,1.2\right]\left(\mathrm{rad}/\mathrm{sample}\right)$ are set to be suppressed by setting $\Gamma \left(q\right)=0$. Figure 3 and Table 2 show that the PSL, MW and SLFOR of these windows are effectively maintained while having steerable sidelobes over designated intervals.

Thus, due to the flexibility of the mask vector and the initial windows, we can design new window functions with steerable sidelobe properties in the frequency domain while maintaining the good properties of the initial windows as much as possible.

### 3.3. Non-Rectangular Applications

As stated in the Introduction, window functions should be specially designed according to specific applications. For instance, the well-known LFM waveform is widely used in radar and sonar systems. However, the rectangle-like spectrum of the LFM yields sinc-like range sidelobes after pulse compression [29]. Thus, windows need to be applied to the kernel of the MF to suppress the range sidelobes. When the spectrum of the LFM is chosen as the initial window, the proposed method, in which the MW, PSL, SLFOR, and steerable sidelobes can be designed, provides more design freedom to control the range sidelobes.

Assuming that the radar-transmitted waveform is an LFM signal, we obtain the echoes of three aerial targets with different radar cross sections (RCSs), the approximate locations of which are known, and additive Gaussian noise is added, the input SNR of which is 30 dB with respect to the strongest echo power. The relative distance between $Target_{0}$ and $Target_{1}$ is approximately 50 bins, and $Target_{2}$ is approximately 300 bins away from $Target_{0}$. However, two of the targets have relatively low RCSs and are covered by the sidelobes of the strong one when using the rectangular window. Thus, we can generate a window with a low sidelobe level over these regions by using the proposed method.

**Figure 4:**
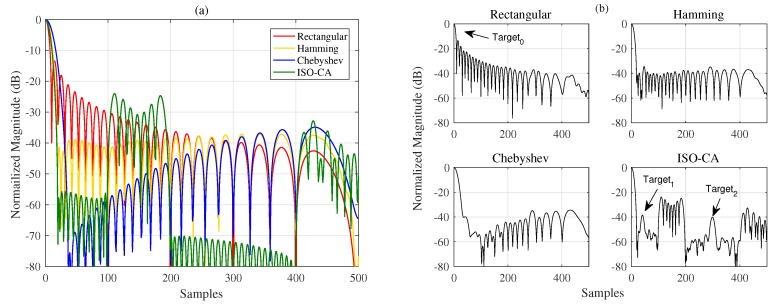
(**a**) Properties of different windows based on a non-rectangular input; (**b**) output of the MF for different window functions.

The characteristics of the spectral windows in the time domain are shown in Figure 4. The original properties of the Hamming and Chebyshev windows are shown to have changed, while the sidelobes of the Chebyshev window function change such that they are no longer equal. In addition, we still cannot see $Target_{1}$ and $Target_{2}$ in Figure 4b using the two traditional windows. However, the window generated using the proposed methods considers the shape of the LFM spectrum. It has low ISRs over the specified regions with a narrow MW. Thus, the three targets can be detected clearly and accurately, as shown in Figure 4b.

### 3.4. ISAR Experiments

**Figure 5:**
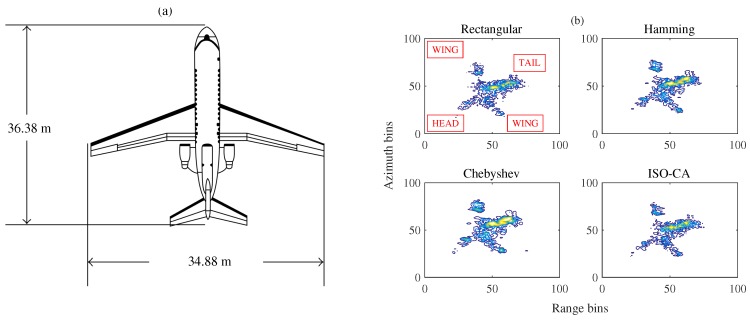
(**a**) Graph of Yak-42; (**b**) ISAR images obtained using different windows (contour plot).

To test the practicability of the proposed method, experiments on some real measured data of a Yak-42 airplane, recorded using a C-band (5.52 GHz) inverse synthetic aperture radar (ISAR) experimental system, are conducted. This system transmits 400 MHz chirp signals with a 25.6 μs pulse width, and the target’s echoes are dechirped and I/Q-sampled at 10 MHz [30,31]. A graph of Yak-42 is presented in Figure 5a.

The FFT is needed for both the range and azimuth focusing to obtain the ISAR image. However, the strong scattering points have high sidelobe levels, and some weak scattering points near the strong ones are easily masked when using a rectangular window. This problem is usually solved by adding Hamming, Chebyshev or other traditional windows to suppress the sidelobes, as shown in Figure 5b, but the effects are not obvious. The traditional window functions have a wide MW and high PSL, as mentioned above, which reduce the ISAR image qualities, including the resolution and the contrast. Considering the proposed methods in this paper, the high PSL should first be suppressed, and the MW should be made as narrow as possible. Then, the wings, head and tail of the plane may be covered by the sidelobes of the strong scatters on the plane body. Thus, we can design the mask vector according to the size of the Yak-42 plane in Figure 5a to suppress the sidelobes over the peak sidelobe region and relative regions among the body, wings, head, and tail of the plane. More scattering points of the plane can be seen by adding the generated windows, as shown in Figure 5b. The image qualities of the ISAR images are also listed in Table 3. Here, we use the MW to represent the resolutions. A lower value denotes a better resolution. Table 3 reveals that the window function generated using the proposed methods yields the best ISAR image result, with a good resolution and high image contrast.

## 4. Results and Discussion

Section 3 presents several simulations and experiments to show the advantages of the proposed ISO-CA including the effectiveness, flexibility, versatility and practicability. First, all the spectral characteristics of the generated window functions, i.e., PSL, MW, and SLFOR, can be controlled. $CA_{1}$ to $CA_{4}$ are the generated windows with different MWs and PSLs. Table 1 shows that the properties of these windows are superior to those of traditional windows. For instance, the MWs of Hanning, Bartlett-Hanning, and $CA_{2}$ are the same, but the PSL of $CA_{2}$ is the smallest. Many similar examples are found in Table 1. Conversely, it can be reasonably inferred that the MW of the CA window is narrower than those of traditional windows when the PSLs are the same. $CA_{5}$ to $CA_{8}$ are the generated windows with a fixed PSL and different SLFORs. The controllable spectral characteristics of the generated windows present the effectiveness of the ISO-CA method. In the meantime, on that basis, steerable sidelobes can be easily added; Figure 3 presents the results if specified regions of the sidelobes should also be suppressed, which shows the flexibility. The versatility is presented by an example in the radar-signal processing field. As stated above, traditional windows do not apply well when the input is non-rectangular like the spectra of the LFM signal. The original spectral characteristics are changed, as shown in Figure 4a. However, the input of the ISO-CA method is defined by designers. Hence, the performance is better than that of the commonly used windows. The shadowing effect between strong and weak targets, which is common in remote sensing sensors, can also be eliminated efficiently by adding steerable sidelobes. To evaluate the practicability, we used measured ISAR data to test the generated CA windows. The ISAR images in Figure 5b show that, compared with the rectangular window, more scattering points of the wings and head of the plane can be seen by adding the Hamming, Chebyshev, and ISO-CA windows. However, the scattering points of the first two windows melt together due to the large MW, which is difficult for further recognition. However, the MW of ISO-CA is close to that of the rectangular window, and steerable sidelobes decrease the shadowing effect of strong scattering points in the plane body. Thus, the ISAR image quality of the ISO-CA, including the resolution and the contrast, is the best.

**Figure 6:**
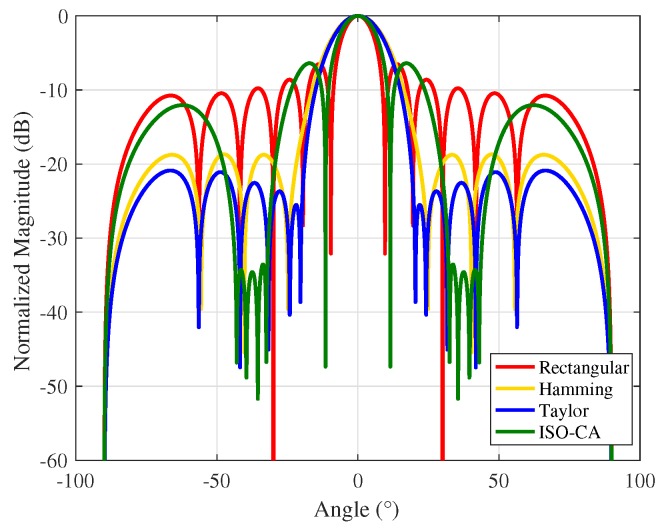
Antenna pattern with different weights.

A window function is a basic signal processing tool that is needed in many sensor signal processing fields such as radar/sonar signal processing. Applications of window functions include spectral analysis/modification/re-synthesis, the design of finite impulse response filters, and beamforming and antenna design. Here, a simple demonstration for antenna design is given in Figure 6. We use the ISO-CA method to suppress the sidelobes within $30^{\circ }$ to $45^{\circ }$ and $-45^{\circ }$ to $-30^{\circ }$ for anti-interference or anti-clutter and at the same time maintain the mainlobe as narrow as possible. Designers can also use the ISO-CA method in other sensor signal processing fields, and we do not list them all. The proposed window functions are substantially generated by solving an optimization problem. The core signal processors of modern sensors are generally the digital signal processor (DSP) and field programmable gate array (FPGA), and the FFT and IFFT are the necessary modules. Since the proposed ISO-CA method for solving the optimization problem contains only the FFT, IFFT, and simple multiplication, it is easy to implement in the DSP or FPGA. In addition, the linear convergence rate guarantees the reliability.

## 5. Conclusions

In this paper, a novel method, i.e., the ISO-CA, for designing general window functions with flexible spectral characteristics is proposed. The matrix multiplication and left inverse calculation in the ISO-CA can be replaced with the FFT and IFFT to improve the efficiency of the iteration. Simulations and experiments show that the generated CA window functions yield better results overall than those of traditional windows and that steerable sidelobes can be acquired both easily and flexibly by designing an appropriate mask vector and stopping criterion. Meanwhile, the favourable properties of the initial windows can be effectively maintained using the proposed methods. Thus, the variation in the initial windows provides more design freedom for specific applications. In addition to the flexibility of ISO-CA, simulations show that the window functions generated by the proposed methods yield better results overall, achieving a narrow MW, a small PSL, and a controllable SLFOR. Experiments based on Yak-42 ISAR real measured data show the practicability of the method, with the ISAR image qualities being greatly improved using the generated window functions.

Although the FFT and IFFT can improve the efficiency of the iteration and the recursion formula is also given in the Appendix, a simple closed-form solution has not been derived so far. The computational complexity will be slightly high if many windows with different lengths are needed at the same time because the length of the window is fixed during the iteration. Generalizing the iteration solution to different lengths is also an interesting topic, and this will be our future work. 

## Figures and Tables

**Table 1:** Property Comparison.

Window	Parameter	MW (×π rad/Sample)	PSL (dB)	SLFOR (dB/oct)
Rectangular	-	0.0137	−13.3	6
Triangular	-	0.0195	−26.5	12
Hamming	-	0.0195	−42.6	6
Hanning	-	0.0215	−31.5	18
Bartlett-Hanning	-	0.0215	−35.9	12
Bohman	-	0.0254	−46.0	24
Blackman	-	0.0254	−58.1	18
Chebyshev	-	0.0254	−80.0	0
Kaiser	β=2.0	0.0137	−18.5	6
	β=3.0	0.0156	−24.0	
	β=4.0	0.0176	−30.3	
Tukey	r=0.25	0.0156	−13.6	18
	r=0.50	0.0176	−15.1	
	r=0.75	0.0195	−19.4	
Gaussian	α=2.5	0.0215	−43.7	6
	α=3.0	0.0234	−56.7	
	α=3.5	0.0273	−71.8	
$CA_{1}$	-	0.0176	−34.0	6
$CA_{2}$	-	0.0215	−49.2	
$CA_{3}$	-	0.0234	−62.6	
$CA_{4}$	-	0.0254	−74.2	
$CA_{5}$	-	0.0156	−19.9	6
$CA_{6}$	-	0.0156	−19.9	12
$CA_{7}$	-	0.0176	−19.9	18
$CA_{8}$	-	0.0195	−19.9	24

**Table 2:** Property Comparison (steerable sidelobes).

Window (Steerable Sidelobes)	${\mathrm{CA}}_{1}$	${\mathrm{CA}}_{4}$	${\mathrm{CA}}_{5}$	${\mathrm{CA}}_{6}$
MW (×π rad/sample)	0.0176	0.0215	0.0156	0.0157
PSL (dB)	−31.7	−46.8	−19.3	−19.8
ISL (interval 1, dB)	−75.6	−86.8	−66.1	−76.9
ISL (interval 2, dB)	−81.8	−92.1	−72.2	−82.3
SLFOR (dB/oct)	6	6	6	12

**Table 3:** Image Quality Comparison.

Image Quality	Rectangular	Hamming	Chebyshev	ISO-CA
Range Resolution	8.07	11.42	15.73	10.47
Azimuth Resolution	5.39	7.49	10.18	6.46
Contrast	4.01	3.79	4.08	5.26

## Abbreviations

The following abbreviations are used in this manuscript:

MW	Mainlobe Width
PSL	Peak Sidelobe Level
SLFOR	Sidelobe Fall-Off Rate
ISO-CA	Inverse of the Shaped Output using Cyclic Algorithm
CA	Cyclic Algorithm
LFM	Linear Frequency Modulation
MF	Matched Filter
DFT/IDFT	Discrete Fourier Transform/Inverse DFT
FFT/IFFT	Fast Fourier Transform/Inverse FFT
RCS	Radar Cross Section
ISAR	Inverse Synthetic Aperture Radar
DSP	Digital Signal Processor
FPGA	field programmable gate array
SVD	Singular Value Decomposition

## Appendix A

To analyze the convergence of the ISO-CA, the relationship between $w_{i}$ and $w_{i+1}$ is derived. Here, we assume that Γ is fixed during the iteration to simplify the derivation, with it being easy to generalize when Γ changes. The *M*-point DFT of $w_{i}$ is:

(A1) $$W_{i}=F_{MN}w_{i}.$$

Then, we have:

(A2) $$W_{\Gamma }=\Gamma ⊙W_{i}.$$

According to the properties of the DFT, the product of two vectors in the frequency domain is equivalent to their circular convolution in the time domain. Thus, we can rewrite (A2) as:

(A3) $$W_{\Gamma }=F_{M}C^{\gamma }{\left[\begin{array}{c} w_{i}\\ 0 \end{array}\right]}_{M\times 1},$$

where:

(A4) $$C^{\gamma }=\left[\begin{array}{cccc} \gamma \left(0\right) & \gamma \left(M-1\right) & \cdots & \gamma \left(1\right)\\ \gamma \left(1\right) & \gamma \left(0\right) & \cdots & \gamma \left(2\right)\\ \vdots & \vdots & \ddots & \vdots \\ \gamma \left(M-1\right) & \gamma \left(M-2\right) & \cdots & \gamma \left(0\right) \end{array}\right]$$

and

(A5) $${\left[\gamma \left(0\right),\gamma \left(1\right),\cdots ,\gamma \left(M-1\right)\right]}^{T}=F_{M}^{-1}\Gamma .$$

Then, we have:

(A6) $$\begin{array}{c} w_{i+1}=F_{MN}^{†}F_{M}C^{\gamma }\left[\begin{array}{c} w_{i}\\ 0 \end{array}\right]=\frac{1}{M}F_{MN}^{H}F_{M}C^{\gamma }\left[\begin{array}{c} w_{i}\\ 0 \end{array}\right]\\ =\frac{1}{M}\left[M\cdot I_{NN},0\right]\left[\begin{array}{cc} C_{NN}^{\gamma } & \cdots \\ \vdots & \ddots \end{array}\right]\left[\begin{array}{c} w_{i}\\ 0 \end{array}\right]\\ =C_{NN}^{\gamma }w_{i} \end{array}.$$

where $C_{NN}^{\gamma }$ is the first *N* rows and *N* columns of $C^{\gamma }$. Assuming that we can obtain the expected spectral characteristics after *x* iterations, we have:

(A7) $$w_{x}={\left(C_{NN}^{\gamma }\right)}^{x}w_{0},$$

where $w_{0}$ is the initial window function. We can rewrite (A7) as:

(A8) $$w_{x}={\left(U_{C}\Sigma_{C}V_{C}^{H}\right)}^{x}w_{0}=U_{C}\Sigma_{C}^{x}U_{C}^{H}w_{0},$$

where $C_{NN}^{\gamma }=U_{C}\Sigma_{C}V_{C}^{H}$ is the singular value decomposition (SVD) and $U_{C}=V_{C}$ because $C_{NN}^{\gamma }={\left(C_{NN}^{\gamma }\right)}^{H}$ according to (A4) and (A5). Then, the rate of convergence is defined as:

(A9) $$\begin{array}{c} \beta =\frac{{∥w_{i+1}\;-\;w_{x}∥}_{2}}{{∥w_{i}\;-\;w_{x}∥}_{2}}\\ =\frac{{∥U_{C}\Sigma_{C}^{i}\left(\Sigma_{C}\;-\;\Sigma_{C}^{x-i}\right)U_{C}^{H}w_{0}∥}_{2}}{{∥U_{C}\Sigma_{C}^{i}\left(I_{NN}\;-\;\Sigma_{C}^{x-i}\right)U_{C}^{H}w_{0}∥}_{2}}\\ =\frac{w_{0}^{H}U_{C}{\left(\Sigma_{C}\;-\;\Sigma_{C}^{x-i}\right)}^{2}\Sigma_{C}^{2i}U_{C}^{H}w_{0}}{w_{0}^{H}U_{C}{\left(I_{NN}\;-\;\Sigma_{C}^{x-i}\right)}^{2}\Sigma_{C}^{2i}U_{C}^{H}w_{0}} \end{array}$$

According to the interlacing theorem for singular values, we obtain:

(A10) $$\sigma_{C}\leq \sum_{n=0}^{M-1}\gamma \left(n\right)=\Gamma \left(0\right)=1,$$

where $\sigma_{C}$ denotes the eigenvalue of $\Sigma_{C}$. Considering the matrix from (A9):

(A11) $$\begin{array}{c} {\left(I_{NN}-\Sigma_{C}^{x-i}\right)}^{-2}\Sigma_{C}^{-2i}{\left(\Sigma_{C}-\Sigma_{C}^{x-i}\right)}^{2}\Sigma_{C}^{2i}\\ ={\left(I_{NN}-\Sigma_{C}^{x-i}\right)}^{-2}{\left(\Sigma_{C}-\Sigma_{C}^{x-i}\right)}^{2} \end{array}$$

the eigenvalue of matrix (A11) can be expressed as

(A12) $$\sigma_{\beta }=\frac{{\left(\sigma_{C}-\sigma_{C}^{x-i}\right)}^{2}}{{\left(1-\sigma_{C}^{x-i}\right)}^{2}},\sigma_{C}\leq 1,$$

By inference, when the items of the mask vector Γ are all ones, we obtain $\sigma_{C}=1$ and thus $\sigma_{\beta }=1$. In this case, $w_{i+1}\equiv w_{i}$. Otherwise, $0<\sigma_{\beta }<1$. According to the generalized Rayleigh quotient, we can obtain from (A9) and (A12) that:

(A13) 0<β<1

Thus, the iteration of the ISO-CA has linear convergence rates [32].
